# Supplementary material for: Performance of the German version of the PARCA-R questionnaire as a developmental screening tool in two-year-old very preterm infants
Source: PLoS One. 2020 Sep 3;15(9):e0236289. doi: 10.1371/journal.pone.0236289 (PMC7470267; doi:10.1371/journal.pone.0236289)
Supplement: S1 Appendix — (PDF) [file pone.0236289.s001.pdf]

The Swiss Neonatal Network and Follow-up Group includes the following local investigators (listed in alphabetical order of study site): Aarau: Cantonal Hospital Aarau, Children's Clinic, Department of Neonatology (Ph. Meyer, C. Anderegg), Department of Neuropaediatrics (A. Capone Mori, D. Kaeppeli); Basel: University Children's Hospital Basel, Department of Neonatology (S. Schulzke), Department of Neuropaediatrics and Developmental Medicine (P. Weber, M. Brotzmann); Bellinzona: San Giovanni Hospital, Department of Pediatrics (G.P. Ramelli, B. Simonetti Goeggel); Berne: University Hospital Berne, Department of Neonatology (M. Nelle), Department of Pediatrics (B. Wagner), Department of Neuropaediatrics (M. Steinlin, S. Grunt); Biel: Development and Pediatric Neurorehabilitation Center (R. Hassink); Chur: Children's Hospital Chur, Department of Neonatology (T. Riedel), Department of Neuropaediatrics (E. Keller, Ch. Killer); Fribourg: Cantonal Hospital Fribourg, Department of Neuropaediatrics (K. Fuhrer); Lausanne: University Hospital (CHUV), Department of Neonatology (J.-F. Tolsa, M. Roth-Kleiner), Department of Child Development (M. Bickle-Graz); Geneva: Department of child and adolescent, University Hospital , Neonatology Units (R. E. Pfister), Division of Development and Growth (P. S. Huppi, C. Borradori-Tolsa); Lucerne: Children's Hospital of Lucerne, Neonatal and Paediatric Intensive Care Unit (M. Stocker), Department of Neuropaediatrics (T. Schmitt-Mechelke, F. Bauder); Lugano: Regional Hospital Lugano, Department of Paediatrics (V. Pezzoli); Muensterlingen: Cantonal Hospital Muensterlingen, Department of Paediatrics (B. Erkert, A. Mueller); Neuchatel: Cantonal Hospital Neuchatel, Department of Paediatrics (M. Ecoffey); St. Gallen: Cantonal Hospital St. Gallen, Department of Neonatology (A. Malzacher), Children's Hospital St. Gallen, Neonatal and Paediatric Intensive Care Unit (J. P. Micallef), Department of Child Development (A. Lang-Dullenkopf); Winterthur: Cantonal Hospital Winterthur, Department of Neonatology (L. Hegi), Social Paediatrics Centre (M. von Rhein); Zurich: University Hospital Zurich (USZ), Department of Neonatology (D. Bassler, R. Arlettaz), University Children's Hospital Zurich, Department of Neonatology (V. Bernet) and Child Development Centre (B. Latal, G. Natalucci).
