# Supplementary material for: Performance of the German version of the PARCA-R questionnaire as a developmental screening tool in two-year-old very preterm infants
Source: PLoS One. 2020 Sep 3;15(9):e0236289. doi: 10.1371/journal.pone.0236289 (PMC7470267; doi:10.1371/journal.pone.0236289)
Supplement: S1 Table — SD, standard deviation; IQR, interquartile range; *, birth weight below 10. percentile; **, above Bell’s stage 2. (PDF) [file pone.0236289.s002.pdf]

	Participants n = 154	Non-participants n = 611	P-value
Gestational age, mean (SD), weeks	29.0 (2.0)	29.3 (2.1)	0.076
Girls, n (%)	79 (51)	285 (47)	0.301
Singletons, n (%)	107 (72)	419 (69)	0.379
Birth weight, mean (SD), grams	1174 (345)	1252 (384)	0.027
z-score, mean (SD)	-.189 (0.818)	-.033 (.845)	0.059
Small for gestational age *, n (%)	12 (9)	51 (8)	0.675
Umbilical artery pH, mean (SD)	7.31 (0.09)	7.30 (0.08)	0.362
10 minutes Apgar score, median (IQR)	8.0 (7.0 to 9.0)	8.0 (7.0 to 9.0)	0.842
Mechanical ventilation, median (IQR), days	1.0 (0.0 to 2.0)	0 (0.0 to 2.0)	0.408
Bronchopulmonary dysplasia, n (%)	15 (11)	59 (10)	0.754
Intraventricular haemorrhage grade ≥ 2, n (%)	4 (3)	22 (4)	0.639
Cystic periventricular leukomalacia, n (%)	3 (2)	14 (2)	0.892
Sepsis, n (%)	9 (6)	65 (11)	0.118
Necrotizing enterocolitis **, n (%)	2 (1)	10 (2)	0.841
Retinopathy of prematurity grade ≥ 3, n (%)	6 (4)	14 (2)	0.265
Length of stay in NICU, days, median (IQR)	47 (30 to 67)	41 (24 to 65)	0.059
Socioeconomic status, median (IQR)	6.0 (4.0 to 6.0)	6.0 (4.0 to 8.0)	0.111
