# Supplementary material for: Performance of the German version of the PARCA-R questionnaire as a developmental screening tool in two-year-old very preterm infants
Source: PLoS One. 2020 Sep 3;15(9):e0236289. doi: 10.1371/journal.pone.0236289 (PMC7470267; doi:10.1371/journal.pone.0236289)
Supplement: S2 Table — N = 153; R, Pearson R coefficient. (PDF) [file pone.0236289.s003.pdf]

PARCA-R scales	Mental Development Index
Non-verbal cognition scale	$R = 0.35, p < 0.001$
Vocabulary sub-scale	$R = 0.54, p < 0.001$
Sentence complexity sub-scale	$R = 0.53, p < 0.001$
Linguistic skill scale	$R = 0.54, p < 0.001$
Parent report composite score	$R = 0.54, p < 0.001$
