# Supplementary figures and images for: Performance of the German version of the PARCA-R questionnaire as a developmental screening tool in two-year-old very preterm infants
Source: PLoS One. 2020 Sep 3;15(9):e0236289. doi: 10.1371/journal.pone.0236289 (PMC7470267; doi:10.1371/journal.pone.0236289)

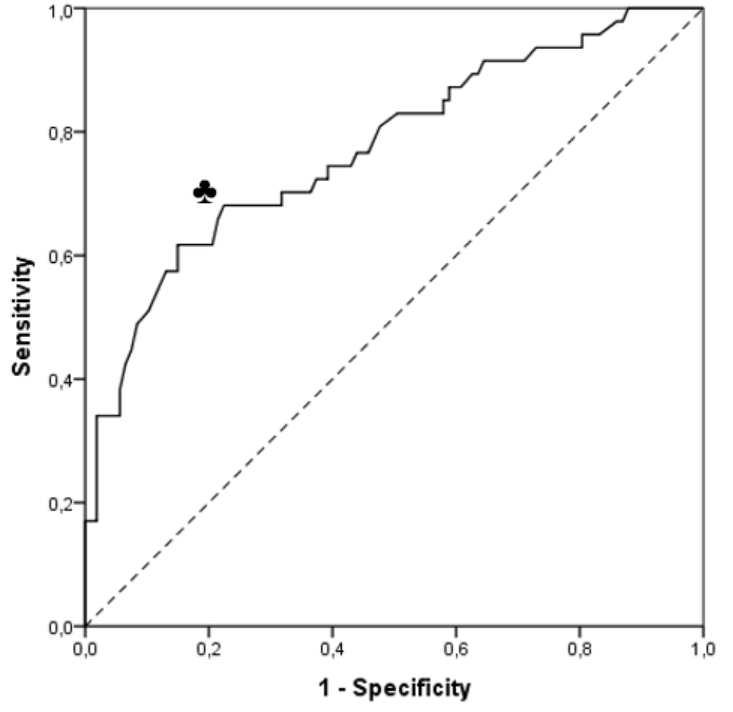

Supplement: S1 Fig — ♣ denotes the cut-off score 63 of the Parent Report Composite (PARCA-r) with the best predictive values for identifying infants with a Mental Development Index < 85. (TIF) [file pone.0236289.s004.tif]

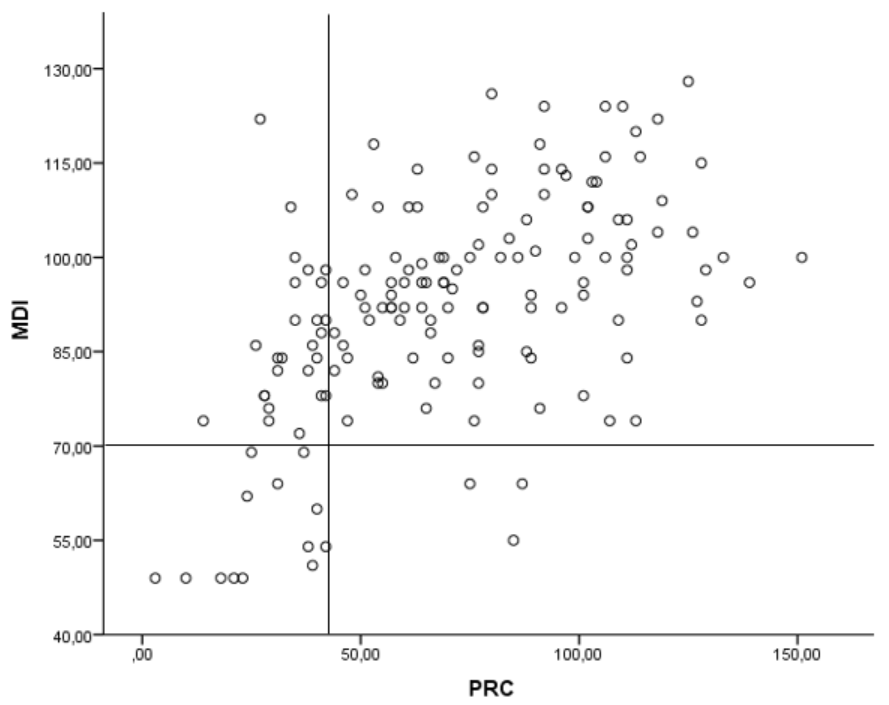

Supplement: S2 Fig — PRC, Parent Report Composite (PARCA-r); MDI, Mental Development Index, Norm (SD), 100 (15), (Bayley’s scales of infant development, 2nd Edition). Vertical line, PRC cut-off score of 44; horizontal line, MDI cut-off score of 70 for defining mental delay. (TIF) [file pone.0236289.s005.tif]
