# Supplementary material for: Performance of the German version of the PARCA-R questionnaire as a developmental screening tool in two-year-old very preterm infants
Source: PLoS One. 2020 Sep 3;15(9):e0236289. doi: 10.1371/journal.pone.0236289 (PMC7470267; doi:10.1371/journal.pone.0236289)
Supplement: S1 Questionnaire — (PDF) [file pone.0236289.s007.pdf]

Elternfragebogen zur Entwicklung extrem frühgeborener Kinder (PARCA-R)

Die Gesundheit und Entwicklung Ihres Kindes im Alter von 2 Jahren

In diesem Formular bitten wir Sie, einige Fragen zu Ihrem Kind und Ihrer Familie zu beantworten. Mit Hilfe Ihrer Antworten wollen wir feststellen, wie Ihr Kind sich im Alter von zwei Jahren entwickelt. Bitte beantworten Sie alle Fragen so genau und vollständig wie möglich.

Wenn Sie beim Ausfüllen des Fragebogens Hilfe brauchen oder Ihnen an den Fragen etwas unklar ist, können Sie bei Ihrem Termin jederzeit Ihren Arzt/Ihre Ärztin dazu befragen.

Dieser Fragebogen wurde mit Genehmigung von Saudino, Dale, Oliver, Petrill, Richardson, Rutter, Simonoff, Stevenson & Plomin (1998) und nach Johnson, Marlow, Wolke, Davidson, Marston, O'Hare, Peacock & Schulte (2004) angepasst. Für Fragen über diese Version vom PARCA-r bitte kontaktieren Sie: Giancarlo.Natalucci@usz.ch, *Swiss Neonatal Network and Follow-up Group*, Schweiz.

WIE IHR KIND SPIELT

Als Mutter oder Vater wissen Sie genau, was Ihr Kind kann und was nicht. Weiter unten finden Sie eine Liste mit verschiedenen Tätigkeiten. Geben Sie bitte an, ob Ihr Kind die jeweiligen Dinge kann oder nicht. Wenn Sie also gesehen haben, wie Ihr Kind die jeweilige Sache macht (oder etwas Ähnliches), dann haken Sie das Kästchen unter „JA“ ab. Wenn Sie wissen, dass Ihr Kind es nicht können würde, dann setzen Sie ein Häkchen unter „NEIN“. Sind Sie sich nicht sicher, ob Ihr Kind eine Tätigkeit machen kann oder nicht, dann haken Sie das Kästchen unter „WEISS NICHT“ ab. Bitte beantworten Sie alle Fragen.

Bitte denken Sie daran, dass diese Fragen für Kinder im Alter von 18 Monaten bis zu 4 Jahren gedacht sind. Einige der Dinge fallen Ihrem Kind vielleicht leicht, andere schwer. Die meisten Kinder im Alter Ihres Kindes können nicht alles, was in der Liste aufgeführt ist.

		JA	NEIN	WEISS NICHT
1	Macht Ihr Kind Ihnen Dinge nach, etwa einen Teddybär an sich drücken? (Wenn Sie nicht sicher sind, probieren Sie es: drücken Sie den Teddy an sich und geben Sie ihn dann Ihrem Kind. Sagen Sie: ‚Jetzt umarm‘ Du den Bär‘	☐	☐	☐
2	Sucht Ihr Kind nach einem Spielzeug, das Sie vor seinen Augen versteckt haben und findet es? (Zum Probieren decken Sie ein kleines Spielzeug mit einem Tuch oder einer Tasse zu und sehen Sie, ob Ihr Kind das Spielzeug aufdeckt)	☐	☐	☐
3	Kann Ihr Kind ein einfaches Teil, wie etwa ein Quadrat oder eine Tierform auf einem Steckpuzzle an die rechte Stelle legen?	☐	☐	☐
4	Es gibt Spielsachen mit mehreren Löchern oder Öffnungen in verschiedenen Formen, etwa einem Kreis, einem Dreieck und einem Stern. Könnte Ihr Kind die Formen in die richtigen Öffnungen einsortieren?	☐	☐	☐
5	Kann Ihr Kind zwei kleine Klötze oder Spielsachen aufeinander stapeln?	☐	☐	☐
6	Kann Ihr Kind ohne Hilfe ein Puzzle zusammensetzen, oder ein ähnliches Spiel, dessen Einzelteile zusammenpassen?	☐	☐	☐
7	Wenn ja, kann es das bei einem Puzzle, das zehn oder mehr Teile hat?	☐	☐	☐
8	Kann Ihr Kind mit der Spitze von einem Wachsmalstift, einem Farbstift oder einer Kreide auf ein Blatt Papier malen?	☐	☐	☐
9	Kann Ihr Kind einen mehr oder weniger geraden Strich auf Papier malen?	☐	☐	☐
10	Blättert Ihr Kind in einem Buch einzelne Seiten um, oder versucht es das?	☐	☐	☐

		JA	NEIN	WEISS NICHT
11	Tut Ihr Kind manchmal so, als wäre ein Gegenstand, zum Beispiel ein Bauklotz, etwas anderes, wie etwa ein Auto oder ein Telefon?	☐	☐	☐
12	Kann Ihr Kind drei kleine Klötze oder Spielsachen ohne Hilfe aufeinander stapeln?	☐	☐	☐
13	Gibt Ihr Kind manchmal im Spiel vor, etwas zu tun? Tut es zum Beispiel so, als ob es auf einem Pferd reitet oder eine Tasse Tee macht?	☐	☐	☐
14	Kann Ihr Kind ein Auto mit den Rädern auf dem Boden entlangschieben?	☐	☐	☐
15	Sieht sich Ihr Kind die Bilder in einem Buch mit Interesse an?	☐	☐	☐
16	Deutet Ihr Kind auf Bilder in einem Buch?	☐	☐	☐
17	Versucht Ihr Kind, Sie nachzuahmen, zum Beispiel, wenn Sie mit einem Löffel in einer Tasse rühren?	☐	☐	☐
18	Kann Ihr Kind sieben kleine Klötze oder Spielsachen ohne Hilfe aufeinander stapeln?	☐	☐	☐
19	Zeigt Ihr Kind, wo Menschen oder Gegenstände sind, wenn Sie fragen: „Wo ist das Licht?“, „Wo ist der Papa?“ oder „Wo ist der Teddybär?“	☐	☐	☐
20	Spielt Ihr Kind je, dass zwei Puppen miteinander spielen, oder miteinander reden, oder dass eine die andere füttert?	☐	☐	☐
21	Spielt Ihr Kind je mit anderen Kindern Rollenspiele, wo es so tut, als wäre es jemand anderer, zum Beispiel Mama, Papa, Polizist oder Krankenschwester?	☐	☐	☐
22	Spielt Ihr Kind je mit anderen Kindern Spiele, bei denen die Kinder abwechselnd an die Reihe kommen?	☐	☐	☐
23	Macht Ihr Kind manchmal Handlungen nach, kurz nachdem es sie beobachtet hat (ein paar Minuten später)?	☐	☐	☐
24	Kann Ihr Kind alleine etwas, zum Beispiel eine Spielsache, aus einem anderen Zimmer holen, wenn Sie darum bitten?	☐	☐	☐
25	Weiss Ihr Kind, wo bestimmte Dinge hingehören, z.B. dass seine Spielsachen in eine Kiste gehören?	☐	☐	☐

		JA	NEIN	WEISS NICHT
26	Kommt es vor, dass Ihr Kind sich von selbst einen Keks (oder Snack) aufspart oder etwas für später beiseitelegt?	☐	☐	☐
27	Haben Sie je gesehen, dass Ihr Kind drei oder mehr Spielsachen zusammen gesammelt hat, bevor es begann, mit ihnen zu spielen?	☐	☐	☐
28	Haben Sie je gesehen, wie Ihr Kind von selbst Dinge (Bauklötze, sonstige Spielsachen) in Gruppen sortiert oder aufgehäuft hat?	☐	☐	☐
29	Holt sich Ihr Kind einen Stuhl oder einen Kasten zum Daraufstellen, wenn es etwas haben will, was außer Reichweite ist?	☐	☐	☐
30	Spricht Ihr Kind beim Telefonieren oder beim Spiel mit einem Telefon in die Sprechmuschel und nicht in den Hörer?	☐	☐	☐
31	Ist Ihr Kind vorsichtig, wenn es einen Becher nach dem Trinken wieder abstellt und versucht es, nichts zu verschütten?	☐	☐	☐
32	Versucht Ihr Kind, an Türknäufen oder Drehverschlüssen zu drehen oder die Deckel von Gläsern auf- und abzuschrauben?	☐	☐	☐
33	Erkennt sich Ihr Kind selbst, wenn es in den Spiegel schaut?	☐	☐	☐
34	Deutet Ihr Kind je mit dem Zeigefinger (dem ersten Finger), auf etwas, wenn es sein Interesse daran zeigen will?	☐	☐	☐

WAS IHR KIND SAGEN KANN

Kinder verstehen wesentlich mehr Wörter, als sie sagen können. Hier interessiert uns nur, welche Wörter Ihr Kind SAGT. Geben Sie bitte alle Wörter an, die Sie von Ihrem Kind gehört haben. Wenn Ihr Kind ein Wort anders ausspricht – z B. „diessen“ für „giessen“ “Nane” für „Banane“ – gilt das auch. Diese Wortliste ist nur eine Auswahl, und Ihr Kind kennt möglicherweise eine Menge anderer Worte, die nicht auf dieser Liste stehen.

Mäh-mäh	Kuetzli (Keks)	Bett	tragen	bleiben
Miau	Saft	Schlafzimmer	jagen	winzig
autsch/au	Fleisch	Couch/Sofa	giessen	nass
oh je/au wei	Milch	Ofen/Herd	fertig/aufhören	nach
wau wau	Erbsen	Treppe	passen	Tag
Bär	Mütze	Fahne	schmusen/kuscheln	heute Abend
Vogel	Kette	Regen	hören	unser
Katze	Schuh	Stern	mögen	die da
Hund	Strumpf/Socke	Schaukel	tun, als ob	dieses
Ente	Kinn	Schule	reissen	wir
Pferd	Ohr	Himmel	schütteln	wo
Flugzeug	Hand	Zoo	schmecken	neben
Schiff	Bein	Freund	lieb/nett	runter
Auto	Kissen	Mama/Mami	denken	unter
Ball	Kamm	Person/Öppe/Mensch	wünschen	Alle
Buch	Lampe/Taschenlampe	Salü/Ciao/Tschüss	Alles weg	viel
Spiel	Teller	Hallo	kalt	könnte
Brot	Müll	Nein	schnell	muss
Fisch	Tablett	Einkaufen	froh	würde
Sauce	Handtuch	Danke	heiss	wenn

WIE IHR KIND SICH AUSDRÜCKT

Wir wüssten gerne, wie Ihr Kind die Wörter verwendet, die es sagen kann. Bitte haken Sie ein Kästchen für jede Frage unten ab, um uns zu sagen, ob Ihr Kind Wörter wie dieses oft, manchmal oder noch nicht verwendet.

Bitte denken Sie daran, dass diese Fragen für Kinder bis zu 4 Jahren bestimmt sind. Viele Kinder im Alter Ihres Kindes werden einige der Wörter oder Sätze hier nicht sagen können.

		OFT	MANCHMAL	NOCH NICHT
1	Spricht Ihr Kind je über vergangene Ereignisse oder über Personen, die nicht anwesend sind? Wenn das Kind zum Beispiel letzte Woche beim Karneval war, könnte es später ‚Karneval', ‚Clown' oder ‚Kapelle' sagen.	☐	☐	☐
2	Spricht Ihr Kind je über künftige Ereignisse? Könnte es etwa ‚Töff-töff' oder ‚Bus' sagen, bevor Sie zu einem Ausflug aus dem Haus gehen, oder ‚Schaukel', wenn Sie zum Spielplatz gehen?	☐	☐	☐
3	Spricht Ihr Kind je über Sachen, die gerade nicht da sind? Fragt es beispielsweise nach einer fehlenden Spielsache, die nicht im Zimmer ist, oder auch nach einer Person, die gerade nicht anwesend ist?	☐	☐	☐
4	Versteht Ihr Kind Sie, wenn Sie nach etwas fragen, was nicht im Zimmer ist? Würde es etwa in sein Zimmer gehen und einen Teddybär holen, wenn Sie es fragen ‚Wo ist der Bär'?	☐	☐	☐
5	Weiss Ihr Kind, wem Dinge gehören? Das Kind könnte beispielsweise auf den Schuh der Mutter deuten und ‚Mama' sagen.	☐	☐	☐
6	Hat Ihr Kind schon begonnen, Worte zusammen zu fügen, wie etwa ‚Papa weg' oder ‚Hund beisst'?	☐	☐	☐

Wenn Sie auf die Frage 6 mit „Manchmal" oder „Oft" geantwortet haben, dann beantworten Sie bitte noch einige letzte Fragen auf der nächsten Seite.

Wenn Sie die Frage 6 mit „Noch nicht" beantwortet haben, können Sie den Fragebogen hier beenden. Vielen Dank, dass Sie sich die Zeit genommen haben. Ihre Antworten sind uns eine grosse Hilfe.

Bitte geben Sie bei jedem SATZPAAR– jeweils A und B – unten an, welches am MEISTEN so klingt, wie Ihr Kind momentan spricht, selbst wenn er oder sie nicht GENAU diesen Satz sagen würde.

Wenn Ihr Kind sogar noch kompliziertere Sätze baut als die hier angegebenen Beispiele, wählen Sie bitte den Satz B.

**7**		(Spricht über etwas, was gerade geschieht)	**8**		(Spricht über etwas, was bereits geschehen ist)	**9**		
A	☐	Turm bauen	A	☐	Papa aufhebt hat	A	☐	Auto meins
B	☐	Ich baue Turm	B	☐	Papa hat mich aufgehoben	B	☐	Das ist mein Auto
**10**			**11**			**12**		
A	☐	Baby weinen	A	☐	Da Hund	A	☐	Kaffee heiss
B	☐	Das Baby weint	B	☐	Da ist ein Hund	B	☐	Der Kaffee da ist heiss
**13**			**14**			**15**		
A	☐	Kann nicht	A	☐	Mag Geschichten	A	☐	Mama Keks
B	☐	Ich kann es nicht	B	☐	Ich mag Geschichten lesen	B	☐	Keks für Mama
**16**			**17**			**18**		
A	☐	Nicht lesen	A	☐	Baby essen	A	☐	Schau her
B	☐	Du sollst das Buch nicht lesen	B	☐	Baby will essen	B	☐	Schau, ich tanze

Vielen Dank, dass Sie sich die Zeit für den Fragebogen genommen haben!
